# Determination of Sphingosine-1-Phosphate in Human Plasma Using Liquid Chromatography Coupled with Q-Tof Mass Spectrometry

**DOI:** 10.3390/ijms18081800

**Published:** 2017-08-18

**Authors:** Emmanuel E. Egom, Ross Fitzgerald, Rebecca Canning, Rebabonye B. Pharithi, Colin Murphy, Vincent Maher

**Affiliations:** 1 Department of Cardiology, The Adelaide and Meath Hospital Dublin, Incorporating the National Children Hospital, Tallaght, 24 Dublin, Ireland; rpharithi@gmail.com; 2 Institute of Technology Tallaght, Blessington Road, Tallaght, 24 Dublin, Ireland; Fitzgerald.ross@gmail.com (R.F.); Rebecca.Canning@postgrad.ittdublin.ie (R.C.); colin.murphy@ittdublin.ie (C.M.); 3 Department of clinical medicine, Education Division, Trinity College Dublin, The University of Dublin, 24 Dublin, Ireland

**Keywords:** high-density lipoprotein (HDL), high-density lipoprotein, liquid chromatography-tandem mass spectrometry (LC-MS/MS), quadrupole time of flight (Q-Tof), liquid chromatography-mass spectrometry (LC-MS), sphingosine-1-phosphate (S1P)

## Abstract

Evidence suggests that high-density lipoprotein (HDL) components distinct from cholesterol, such as sphingosine-1-phosphate (S1P), may account for the anti-atherothrombotic effects attributed to this lipoprotein. The current method for the determination of plasma levels of S1P as well as levels associated with HDL particles is still cumbersome an assay method to be worldwide practical. Recently, a simplified protocol based on liquid chromatography-tandem mass spectrometry (LC-MS/MS) for the sensitive and specific quantification of plasma levels of S1P with good accuracy has been reported. This work utilized a triple quadrupole (QqQ)-based LC-MS/MS system. Here we adapt that method for the determination of plasma levels of S1P using a quadrupole time of flight (Q-Tof) based LC-MS system. Calibration curves were linear in the range of 0.05 to 2 µM. The lower limit of quantification (LOQ) was 0.05 µM. The concentration of S1P in human plasma was determined to be 1 ± 0.09 µM (*n* = 6). The average accuracy over the stated range of the method was found to be 100 ± 5.9% with precision at the LOQ better than 10% when predicting the calibration standards. The concentration of plasma S1P in the prepared samples was stable for 24 h at room temperature. We have demonstrated the quantification of plasma S1P using Q-Tof based LC-MS with very good sensitivity, accuracy, and precision that can used for future studies in this field.

## 1. Introduction

Atherosclerotic cardiovascular disease (ASCVD) is the leading cause of death worldwide [1,2]. On the basis of 2012 mortality rate data, more than 48,000 people die of ASCVD each day, an average of one death every 1–2 s [2]. The cause of ASCVD is multifactorial, and may include factors related to lifestyles, age, male gender, and other factors such as arterial hypertension, type 2 diabetes, and dyslipidaemia [3]. Dyslipidaemias are disorders of lipid and/or lipoprotein metabolism that may result in high total cholesterol, high low density lipoprotein (LDL) cholesterol, high triglycerides, and low high density lipoprotein (HDL) cholesterol. Several epidemiological studies reported an inverse relationship between the levels of HDL-cholesterol and the presence or development of ASCVD [4,5]. The incidence of ASCVD events in a normal population appears to be inversely related to the concentration of serum HDL-cholesterol, with low levels being associated with increased ASCVD risk [6,7]. Based upon data from the Framingham Heart Study, the risk for ASCVD increases by about 25 percent for every 5 mg/dL (0.13 mmol/L) decrement in serum HDL-cholesterol below median values for men and women [7]. HDL-cholesterol levels are also predictive of coronary events in patients with known ASCVD. These observations have stimulated several laboratories and research groups worldwide to focus their research efforts on the development of therapies aiming to increase HDL-cholesterol.

Despite the evidence in support of a potential benefit from raising HDL-cholesterol, accumulating evidence suggests that simply increasing the amount of circulating HDL-cholesterol does not protect against ASCVD [8,9]. This led to the hypothesis that HDL in some patients may be dysfunctional and its other properties and compositions might be more important than its cholesterol cargo [10,11,12]. In fact, the cholesterol component is only one of many structural or functional components of the HDL particle, which in addition consist of several molecules of apoA-I, phosphatidylcholine molecules, minor proteins, diverse lipids species including sphingosine-1-phosphate (S1P), and even microRNAs [10,13,14,15,16]. Thus, if HDL-cholesterol is found not to be causally associated with ASCVD, it is possible that other components of HDL, particularly S1P, may directly or indirectly contribute to the anti-atherothrombotic potential of HDL [17].

Several studies [18,19,20] have demonstrated that HDL may serve as a carrier for S1P, a lysosphingolipid exerting its biological actions mainly via the binding to five G protein-coupled receptors, named S1P1-5 [21,22,23]. S1P has been shown to regulate several biological actions in a variety of different organ systems, including the cardiovascular system [24,25]. Accumulating evidence suggests that S1P may mediate several actions of HDL including vasodilation, angiogenesis and endothelial barrier function, and protection against atherosclerosis and ischaemia/reperfusion injury [10,11,12]. There is therefore an urgent and unmet need to investigate the levels of S1P in HDL particles (S1P-rich HDL) from different populations, particularly those with ASCVD. Such a study would not only clarify whether changes in S1P-rich HDL may explain the commonly observed HDL dysfunction, which is typical in patients with ASCVD, but would also elucidate whether S1P-rich HDL may serve as a potential diagnostic and therapeutic target for the treatment and prevention of ASCVD.

The clinical value of S1P-rich HDL concentrations is dependent on developing a robust and inexpensive assay for the quantification of S1P in plasma. Liquid chromatography-tandem mass spectrometry (LC-MS/MS) is currently the best technology in terms of sensitivity, quantitative precision, and throughput capabilities for such analyses in small samples [26,27,28,29,30,31]. Recent work by Frej and co-workers [32] describes an LC-MS/MS method for the quantitation of S1P. This work involves relatively simple sample preparation and addresses both sample handling and chromatography challenges associated with the analysis of S1P. The objective of this study was to determine if we could adapt the methodology described by Frej et al. [32] to quantify S1P in plasma using a Q-Tof-based LC-MS system, with a view to using it for further studies. Here we optimize and demonstrate the quantitation of S1P with very good sensitivity, accuracy, and precision using Q-Tof LC-MS.

## 2. Results

### 2.1. Optimising Chromatography

**Figure 1:**
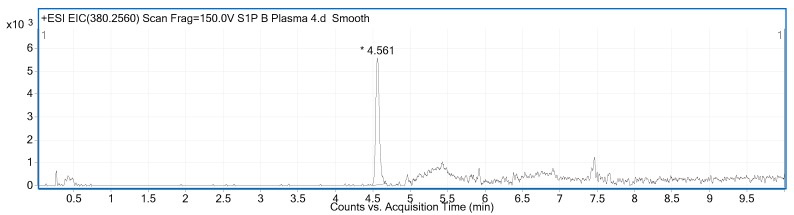
Representative extracted ion chromatogram (EIC) at *m*/*z* 380.2560 corresponding to the [M + H]^+^ ion of sphingosine-1-phosphate (S1P). S1P elution time and peak shape are illustrated. The retention time corresponds to that determined for the standards.

When assessing methanol extract from human plasma, S1P eluted after 4.6 min with very good peak shape (Figure 1). The S1P molecule contains a long hydrophobic chain and hydrophilic phosphate group conferring S1P amphipathic properties [33]. Furthermore, S1P has an amino group as well as a hydroxyl group conferring S1P zwitterionic properties [34]. These characteristics are important to consider when performing chromatographic separations of S1P as secondary interactions within the column and LC system can have detrimental effects on peak shape and carryover. The previously described work addressed both carryover and peak shape and through careful column and mobile phase selection excellent peak shape and low carryover were obtained [32].

**Figure 2:**
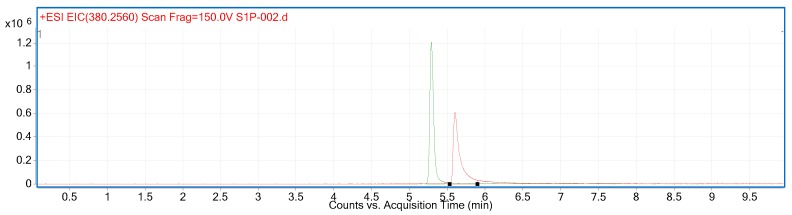
Comparison between the Waters X-Select CSH column, as used previously [32] (Red), and a Phenomenex Kinetex EVO C18 used here (Green).

With our system, we observed better peak shape with a different column. Figure 2 shows a comparison between the Waters X-Select CSH column, as used by Frej and colleagues (Red) [32], and a Phenomenex Kinetex EVO C18 (Green). The stationary phase of both columns is reported to carry a slight positive charge. In acidic mobile phase, both S1P and the column stationary phase will be positively charged and the repulsive forces between them reduce the occurrence of secondary polar interactions and hence result in better peak shape. The column used here has smaller (1.7 V 2.5 µM) and superficially porous particles. Both factors are likely to contribute to increased efficiency. This column is used throughout the remainder of this study.

Although 0.1% formic acid produced a stronger analyte response with our system than 1.0% formic acid used in the previous work [32], otherwise the mobile phase and gradient composition were unchanged.

### 2.2. Compound Identification and Selectivity

**Figure 3:**
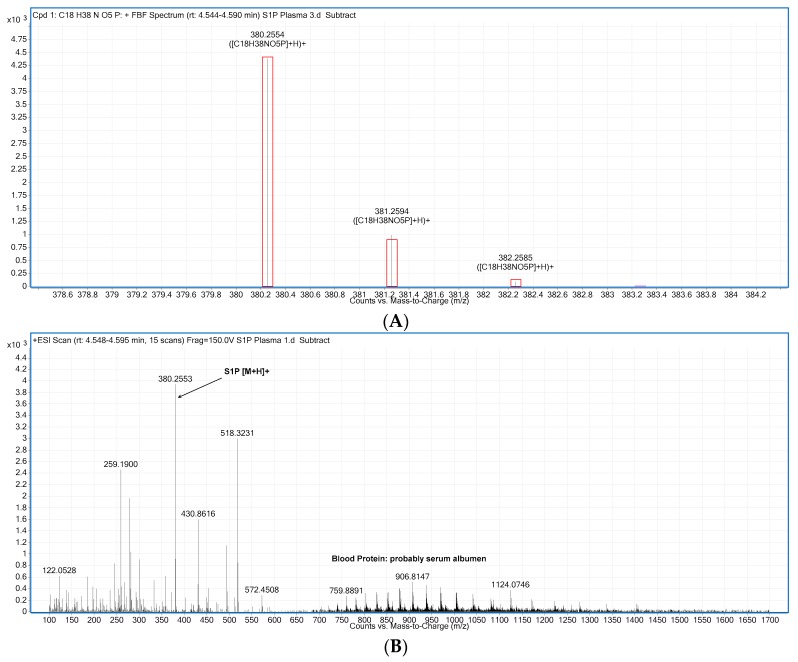
(**A**) When only the ions of interest are extracted [M+H]^+^, both mass accuracy and isotope pattern are consistent with the presence of S1P. The black lines are the spectrum overlaid with predicted isotope pattern; (**B**) Representative raw spectrum extracted under the S1P peak with background subtracted. The evidence of blood proteins (probably serum albumin) co-eluting is shown by the peaks in the charge envelope between ~700 and 1500 *m*/*z*.

A signal for S1P [M + H]^+^ in plasma was observed with the correct *m*/*z* 380.2560 at the same retention time observed for spiked standards. As the Q-Tof is a high resolution, accurate mass instrument (mass accuracy typically better than 2 ppm on the instrument used here) observed ions are much more likely to have a specific molecular formula and the possibility of false positives is considerably reduced. Furthermore, the isotope peaks are also detected and if they display the correct pattern of spacing and abundance this is further evidence that the signal corresponds to the correct molecular formula. As illustrated in Figure 3A, the mass accuracy and isotope pattern are very good matches for S1P. The red overlay is a model of the expected isotope pattern for S1P and matches the observed spectrum. (This identification is done using the Find by Formula function in the Agilent Masshunter Qualitative data analysis software). While [M + H]^+^ is the predominant ion corresponding to S1P, the presence of the small amounts of the corresponding sodium adduct [M + Na]^+^ also support the identification. Quantitation is based on the [M + H]^+^ signal.

The evidence of blood proteins (probably serum albumen) co-eluting is shown by the peaks in the charge envelope between ~700 and 1500 *m*/*z* (Figure 3B).

### 2.3. Calibration Curves

Thirty nanomolar (30 nM) internal standard (d7-S1P) in methanol was used as the precipitation solution to prepare all samples and standards. Standards at 10 concentration levels (0.0016 to 4.5 μM) were prepared in 4% BSA and the samples for injections prepared and analyzed in exactly the same way as the plasma samples. No signal above baseline was observed for levels 1 and 2 (Table 1) and level 3 was not consistently detected. The calibration curve was therefore created starting from the level 4 standard (0.05 µM). As this concentration could be measured with accuracy and precision >10% it was also determined to be LOQ.

Three injection replicates of levels 4 to 10 were plotted and a least squares regression line (Figure 5) with *R*^2^ of 0.9966 was obtained. Visual inspection shows the data to be heteroscedastic with the absolute variance increasing at higher concentration. The plot of the positive and negative standard deviation against calibration levels show this is the case (Figure 6).

Linear least square regression requires data to be homoscedastic so that increased variance at the higher concentrations does not exert a disproportionate influence on the variance of the curve as a whole such that it is less accurate at the lower levels [35]. Heteroscedastic data can be better represented using a weighted least squares regression [35]. Weighted calibration curve models were investigated and found to improve accuracy and precision at lower concentrations.

As determination of variance of the regression model requires that the data be homoscedastic, estimation of the limits of detection (LOD) and the limit of quantification (LOQ) from the slope and variance of the calibration curve (LOD = 3.3 × σ/slope and LOQ = 10 × σ/slope) will not be reliable and should be performed experimentally [36]. LOD and LOQ are determined as a function of the precision and accuracy within specified limits, as presented below.

### 2.4. Calibration Model Selection

Non-weighted and 1/*x* weighted linear calibration curves were compared. 1/*x* weighted models were found to give improved accuracy and precision, at lower concentrations over the unweighted model (Table 1 and Table 2 summarized the data obtained using 1/*x* weighted model).

1/*x* weighting was the simplest model that provided the best fit of the data points and no benefit was seen by using higher order weighting models (data not shown). Using the weighted models, the concentration of the level 4 standard (0.05 µM) could be determined with 90–110% accuracy and precision ≤10%, fulfilling criteria for LOQ. LOD can be estimated to be 0.016 µM (Lower Limit of Quantification (LLOQ)/3.3). Without weighting acceptable precision and accuracy were not achieved below level 5 (0.15 µM).

**Figure 4:**
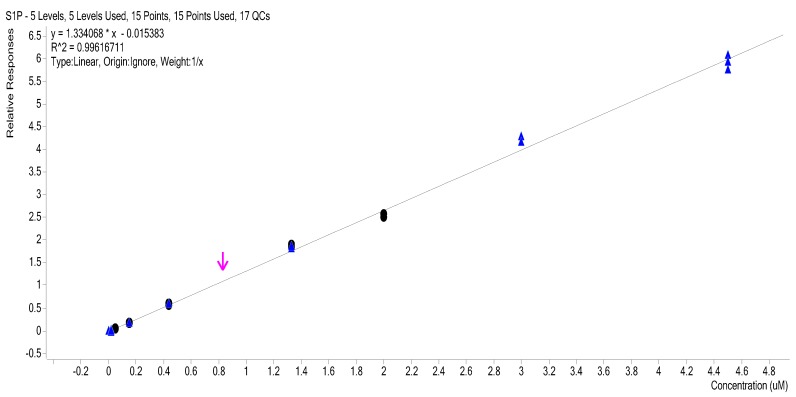
1/*x* weighted calibration curve recommended to be used. Black dots: calibration standards; blue triangles: QC samples; pink arrow: denotes human plasma. Black dots represent calibration standards; blue triangles are QC samples and pink arrow is a human plasma sample.

**Figure 5:**
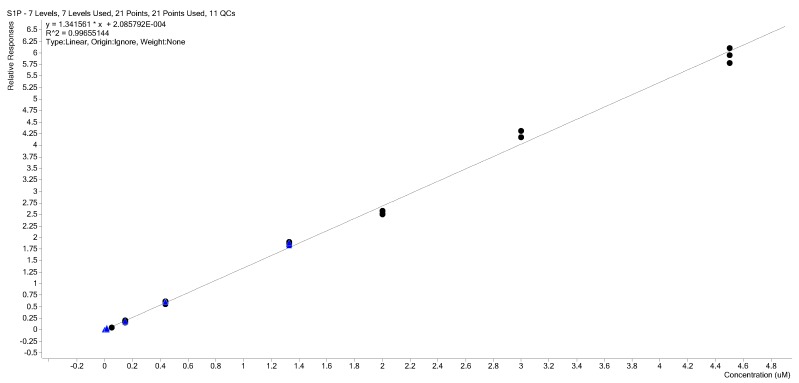
Non-weighted linear regression calibration curve of three injection replicates at seven calibration levels (4 to 10) (0.05 to 4.5 µM) with very good correlation and linearity over the entire range. The heteroscedasticity of the data is clear with absolute variance increasing with concentration. Black dots represent calibration standards; blue triangles are QC samples.

**Figure 6:**
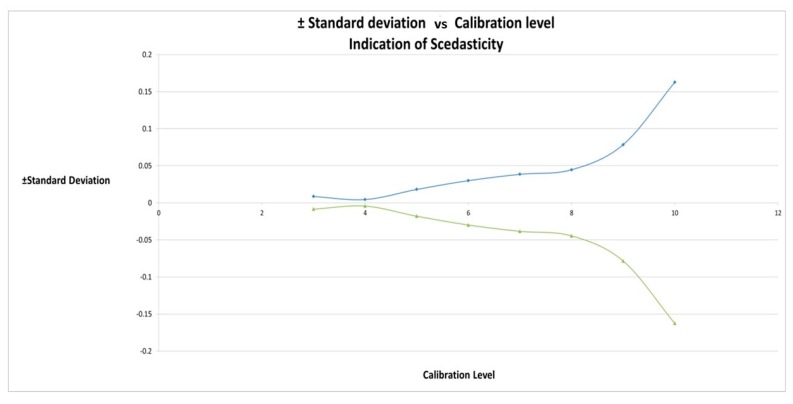
The data is heteroscedastic and would be better modelled using a weighted linear regression. Plot of the positive and negative standard deviation against calibration levels show increased absolute variation with increasing concentration.

The calibration curve used in this method is a 1/*x* weighted linear regression S1P:d7-S1P (internal standard) response ratio versus S1P concentration (Figure 4). The curve ignores the origin and is based on data from three injection replicates at five calibration levels ranging from 0.05 to 2.0 µM S1P in 4% BSA that were prepared and analyzed exactly the same as the plasma test samples.

### 2.5. Range

The range of the method is 0.05 to 2 µM. Although the response was shown to be linear up to the highest level investigated (4.5 µM), and it was observed that the inclusion of calibration standards to this level increased the correlation coefficient (*R*^2^ = 0.9977), it was not envisaged that quantitation of concentrations above 2 µM would be required. Because of the heteroscedastic nature of the data, calibration levels above 2 µM are not included so they cannot exert unwanted influence on the variability of the model as a whole and reduce its performance at lower levels. Even without their inclusion in the calibration curve, the method could quantify at levels above 2 µM with excellent accuracy and precision (Table 1, QC levels 9 and 10).

**Figure 7:**
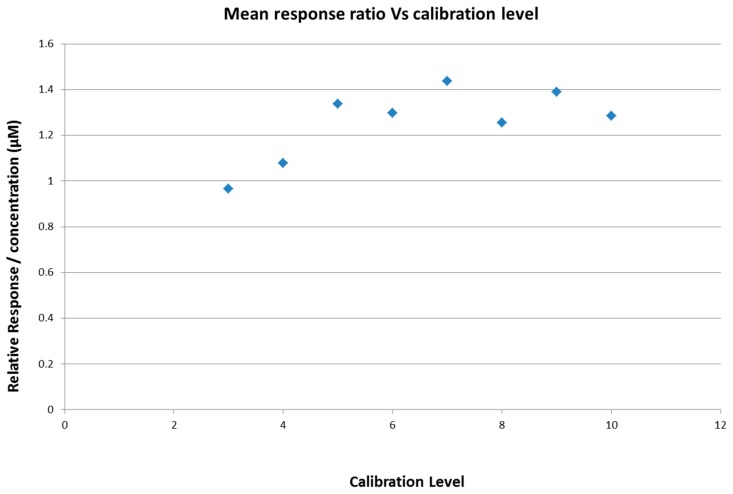
Response/concentration ratio is constant from 1.5 to 4.5 µM with a decline noticeable at 0.05 µM.

Figure 7 indicates that the response to concentration remains constant between levels 5 and 10 and begins to decrease at level 4 (0.05 µM). However, as accuracy and precision remain acceptable, 0.05 µM is the lower level of quantitation (LLOQ) (as discussed below).

The concentration of a human plasma sample was determined to be 1 µM ± 0.09 (*n* = 6), showing that the calibration range of the method is suitable for analysis of S1P in plasma (Table 3). Four different plasma samples were prepared and each was injected at three different time points. This was in good agreement with the observations of Frej et al. [32].

### 2.6. Accuracy and Precision

The average accuracy over the stated range of the method was found to be 100 ± 5.9% with precision at the LOQ better than 10% when predicting the calibration standards. When using separate QC standards, the concentration was determined with accuracy and precision of 104.5 ± 3.8%.

### 2.7. Estimated Lower Level of Detection (LLOD) and LLOQ

LLOD and LLOQ were estimated to be 0.016 µM and 0.05 µM, respectively, based on analysis of the calibration data. The LOQ was estimated as the lowest calibration level that could be determined with better than 90–110% accuracy and precision ≤10%, LLOD was estimated as (LLOQ/3.3) 0.016 µM. S1P at this level was detected but not quantified with good accuracy or precision.

Using signal to noise ratios to determine LOD and LOQ was not deemed appropriate for this data due to the difficulty of reliably defining the noise, the level of which can be an artefact of how the sample is acquired and processed.

As mentioned above, the requirement for homoscedastic data precluded the possibility to estimate LOD and LOQ from the slope and variance of the calibration curve (LOD = 3.3 × σ/slope and LOQ = 10 × σ/slope).

### 2.8. Sample and Instrument Stability over Time

**Figure 8:**
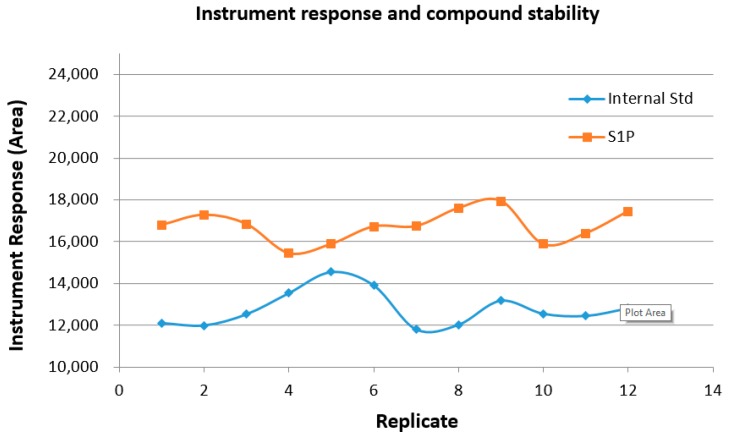
The consistency of the signal over greater than 12 h and 50 injections demonstrates the stability of the instrument and the samples. (Quality control and calibration injections were performed between the above replicates).

The response of S1P and internal standard of the plasma samples was monitored and remained relatively constant (the coefficient of variation (CV), also known as relative standard deviation (RSD), was 4.31% and 6.41%, respectively) over the course of the analysis (>12 h). Between each analysis calibration standards, QC standards, and blanks were run (>50 injections). This shows that the instrument sensitivity and sample sensitivity were stable for at least this time after sample preparation and this number of injections (Table 3, Figure 8). It was observed that plasma samples stored in the autosampler at ambient temperature produced the same instrument response for S1P when reanalyzed after 24 h (data not shown).

During analysis of patient samples monitoring of internal standard response and analysis of QC samples throughout the batch should be employed to ensure the method was performing over the course of the analysis.

### 2.9. Injection Carryover

In solvent blanks injected after the high level calibration standards, no signal for S1P larger than baseline was observed.

## 3. Discussion

We have demonstrated a selective LC-MS protocol for the quantification of plasma levels of S1P, using d7-S1P as internal standard based on existing validated methodology but using a different mass spectrometer (Q-Tof instead of triple quadrupole (QqQ)). In comparison to the method on which this work is based, less absolute sensitivity was achieved. This was expected, as collecting data by MRM on a QqQ MS/MS is typically the most sensitive MS technique for quantitative analysis. However, absolute sensitivity is not of paramount importance here, as physiological levels of S1P are well above LOQ using either method. The ability to determine this with good selectivity accuracy and precision is more important.

Both methods offer excellent specificity. QqQ LC-MS/MS confirms identity by retention time and detecting product ions from a particular precursor. Q-Tof identifies ions by high resolution accurate mass and can perform LC-MS/MS for further confirmation.

One advantage of acquiring Q-Tof data is that it collects data for all *m*/*z* values, not just the analytes of interest. This offers the possibility (subject to ethical approval) of comparing patient populations and performing differential analysis by reinterrogation of existing data without having to rerun samples.

This quantitative assay has no carryover above detectable limits and shows some enhancements on previously published methods [32,37,38,39,40]. The average accuracy over the stated range of the method was found to be 100 ± 5.9%, with precision at the LOQ better than 10% when predicting the calibration standards. Using separate QC standards, concentration was determined with accuracy and precision of 104.5 ± 3.8%. LLOD and LLOQ were estimated to be 0.016 and 0.05 µM, respectively, based on analysis of the calibration data. The LOQ was estimated as the lowest calibration level that could be determined with better than 90–110% accuracy and precision ≤10%; LLOD was estimated as (LLOQ/3.3) 0.016 µM. The concentration of a human plasma sample was determined to be 1 ± 0.09 µM, showing that the calibration range of the method is suitable for analysis of S1P in plasma. These values of S1P are in good agreement with previous reports [26,27,28,29,31,32,36,37,38,39,40,41].

Using signal to noise ratios to determine LOD and LOQ was not deemed appropriate for this data due to the difficulty of reliably defining the noise, the level of which can be an artefact of how the sample is acquired and processed. As mentioned above, the requirement for homoscedastic data precluded the possibility to estimate LOD and LOQ from the slope and variance of the calibration curve (LOD = 3.3 × σ/slope and LOQ = 10 × σ/slope) [36]. A better resolution and peak shape were also observed using a smaller particle superficially porous particle-based column, which ultimately increases the sensitivity of the method.

Despite enhanced chromatographic performance, one drawback to using smaller particle columns is that they can get blocked easier. During initial method development, increased back pressure, retention time drift, and loss of sensitivity was observed when injecting undiluted 4% BSA standards. Phospholipids and proteins from blood based samples are well known culprits for column contamination [42]. As this study is looking at sphingolipids, more extensive sample preparation is not likely to be helpful, as the aim of most clean up procedures is to remove phospholipids from the sample. Using a guard column and monitoring retention time and system pressure to inform when the guard should be changed was considered. However, preparation of the calibration standards by dilution in tris-buffered saline (TBS) in the same manner outlined for the plasma samples does appear to reduce or prevent increases in back pressure and retention time drift [43]. Also, as shown above, instrument sensitivity has been unchanged over the course of >50 consecutive injections run over >12 h. This indicates stability of the instrument and the samples. Furthermore, it was observed that plasma samples stored in the autosampler at ambient temperature produced the same instrument response for S1P when reanalyzed after 24 h.

In summary, with some minor modifications of the method published by [32], we have been able to optimize and adapt the LC-MS/MS QqQ protocol for quantifying plasma S1P with very good sensitivity, accuracy, and precision for use on the LCMS Q-Tof in our laboratory. The modifications included the use of a different column resulting in an improved chromatography; the use of 0.1% instead of 1% formic acid, which also resulted in an improved instrument response without detriment to the chromatography; and finally, the dilution of the 4% BSA calibration standards in concentrated Tris-buffered saline resulting in increased sensitivity due to matrix dilution. The method overall shows very good sensitivity, accuracy, and precision over a range of concentrations that S1P may be expected to be found at in human plasma. The results presented are a verification of the adapted literature method. This paper should, therefore, be used as a reference paper for future studies in this field.

## 4. Materials and Methods

### 4.1. Study Population and Samples Preparation

This study complied with the Declaration of Helsinki and was approved by the local research ethics committees (Adelaide and Meath Hospital, Dublin, incorporating the National Children’s Hospital, Tallaght, Ireland), and all participants gave written informed consent before entry. Blood samples were obtained from six male healthy volunteers aged 40 to 55 years-old without any significant past medical history. Venous blood samples were obtained from the antecubital vein using a minimum 21-guage needle. Blood samples were immediately dispensed into 3 mL ethylenediaminetetraacetic acid (EDTA) tubes with 2-chloroadenosine (0.05 mmol per liter) and procaine hydrochloride (0.154 mol per liter) and equilibrated at 4 °C. All blood samples collected into EDTA with 2-chloroadenosine and procaine were centrifuged at 3300× *g* for 15 min at 4 °C. Aliquots of plasma (0.5 mL) were stored at −80 °C.

LC-MS determinations of S1P levels were essentially performed as per previously published methods [32] and adapted to the LC-MS Q-Tof instrumentation available to us. The 30 nM internal standard solution (deuterium-marked S1P (d7-S1P)) in methanol was used as the precipitation solution to prepare all samples and standards. Calibration standards were prepared by the dilution of stock solution in 4% bovine serum albumin (BSA) to obtain the desired concentrations. Standards at 10 concentration levels (0.0016 to 4.5 µM) were used for initial studies. Subsequently five calibration levels were used (0.5, 0.15, 0.44, 1.33, 2.0 µM). Calibration and quality control (QC) standards were prepared and analyzed in exactly the same way as outlined for the plasma samples below. Solutions containing 0.15, 0.44, and 1.33 µM of S1P were used as QCs.

Plasma samples (10 µL) were diluted in tris-buffered saline (TBS) (55 µL) and prepared for analysis by protein precipitation with ice cold methanol (200 µL) containing internal standard (concentration 30 nM). The samples were kept in an ice bath for 20 min then centrifuged at 1300 rpm for two minutes. One hundred fifty microliters (150 µL) of the supernatant was transferred to a polypropylene LC-MS vial and analyzed by LC-MS (Q-Tof).

### 4.2. Chemical, Reagents, and Instrumentation

S1P (d-erythro-sphingosine-1-phosphate) was purchased from Caymen chemicals (Cambridge Bioscience, Cambridge, UK) and d7-S1P was purchased from Avanti Polar Lipids (Stratech, Newmarket, UK). Formic acid, TBS, and essentially fatty acid and globulin-free bovine serum albumin (BSA), were purchased from Sigma-Aldrich (Tallaght, Dublin, Ireland). Methanol and acetonitrile were of LC-MS grade (Fisher-Optima, Dublin, Ireland) and Acetone HPLC grade (Romil/Lennox, Dublin, Ireland).

Waters X-Select CSH 2.5 µM, (2.1 mm × 50 mm) and Phenomenex Kinetex EVO C18 1.7 µM, (2.1 mm × 50 mm) columns were compared and the latter was ultimately selected and used for the work described here.

The LC-MS system consisted of an Agilent 1260 series HPLC system coupled to an Agilent 6530 Q-Tof mass spectrometer equipped with dual ESI ion source. Data was acquired, analyzed, and calculated using the LC-MS data system software (Masshunter B.06.00, Agilent, Santa Clara, CA, USA).

S1P was separated using a gradient of A (water/methanol/formic acid 97/2/0.1 (*v*/*v*/*v*)) and B (methanol/acetone/water/formic acid 68/29/2/0.1 (*v*/*v*/*v*/*v*)) flow rate of 0.4 mL/min: 0.00 min 50% B, 5.40 min 100% B, 5.40–8.90 min 100% B, 9.00–10.00 min 50% B. The column was maintained at 60 °C.

The Q-Tof MS was operated in 2 GHz, extended dynamic range mode and data was acquired in MS positive mode at five scans per second across a *m*/*z* range of 100–1700. Source parameters: Drying gas 350 °C, 12 L/min; Nebulizer 35 psig; VCap 3500 V; Fragmentor 150 V. Mass reference ions *m*/*z* 121.0509 and *m*/*z* 922.0098 were continually infused via the reference nebulizer (10 psig).

### 4.3. Method Validation

#### 4.3.1. Stability and Selectivity

Stability and selectivity were assessed using six independent blank human plasma samples. The chromatograms of blank human samples were subsequently compared with the corresponding spiked plasma for the test of endogenous interferences.

#### 4.3.2. Carryover

After determination of the highest calibration sample, a blank sample was injected (mobile phase A or TBS buffer) and the S1P peak calculated. The degree of carryover was assessed by dividing the S1P peak levels in the blank sample with that of the calibration sample from five independent experiments [44].

#### 4.3.3. Linearity, Precision, and Accuracy

The linearity of each calibration curve was analyzed by plotting the ratio of the peak areas of the analyte and internal standard (*y*) against the spiked levels of S1P (*x*) [32]. The correlation coefficient (*R*^2^) was calculated automatically for each calibration curve by the LCMS data analysis software, Agilent Masshunter Quantitative Analysis B.06.

The precision and accuracy of the method were assessed by analysis of the QC samples within runs (intra-day precision and accuracy). Accuracy was expressed as the mean of measured concentrations/spiked concentrations × 100, and precision was determined using the coefficient of variation CV % as standard deviation/mean of measured concentration × 100 [45].

## Figures and Tables

**Table 1:** Summary of calibration data showing accuracy and precision. Mean accuracy = 100.0 ± 5.9. Levels 4 to 8 are calibration standards (bold) from which the curve (Figure 4) was built. Green shading denotes 90–110% accuracy or <10% precision (RSD), red shading precision or accuracy outside this specification. Three injection replicates at each level.

Level		1/*x* Weighted Curve Levels 4–8 3 Replicates (R2: 0.9962)						
	Known Conc		Calculated Conc	Accuracy	Conc Mean	Mean Accuracy	Conc SD	Precision
	(μM)		(μM)	%	(μM)	%	(μM)	RSD %
1	0.0016		0.0141	883.8				
2	0.005							
3	0.016		0.014	87.7				
3	0.016		0.036	225.2				
3	0.016		0.05	312.5				
3	0.016		0.0231	144.4	0.036	157.43	0.011	30.21
**4**	**0.05**	**Cal**	**0.0453**	**90.7**				
**4**	**0.05**	**Cal**	**0.0482**	**96.4**				
**4**	**0.05**	**Cal**	**0.0519**	**103.8**	**0.048**	**93.38**	**0.003**	**5.57**
**5**	**0.15**	**Cal**	**0.1352**	**90.1**				
**5**	**0.15**	**Cal**	**0.1523**	**101.5**				
**5**	**0.15**	**Cal**	**0.1619**	**107.9**	**0.150**	**92.53**	**0.011**	**7.37**
**6**	**0.44**	**Cal**	**0.47**	**106.8**				
**6**	**0.44**	**Cal**	**0.4262**	**96.9**				
**6**	**0.44**	**Cal**	**0.4393**	**99.8**	**0.445**	**101.34**	**0.018**	**4.12**
**7**	**1.33**	**Cal**	**1.3863**	**104.2**				
**7**	**1.33**	**Cal**	**1.4165**	**106.5**				
**7**	**1.33**	**Cal**	**1.4439**	**108.6**	**1.416**	**98.04**	**0.024**	**1.66**
**8**	**2.00**	**Cal**	**1.8894**	**94.5**				
**8**	**2.00**	**Cal**	**1.9494**	**97.5**				
**8**	**2.00**	**Cal**	**1.894**	**94.7**	**1.911**	**100.89**	**0.027**	**1.43**
9	3.00		3.1447	104.8				
9	3.00		3.2439	108.1				
9	3.00		3.1393	104.6	3.176	101.17	0.048	1.51
10	4.50		4.4759	99.5				
10	4.50		4.5893	102				
10	4.50		4.3456	96.6	4.470	102.87	0.100	2.23

**Table 2:** Analysis of Quality Control (QC) standards. Mean accuracy = 104.5 ± 3.8. These values are from separate QC samples. Green shading denotes 90–110% accuracy or <10% precision (RSD). Two injection replicates at each level.

Level	Known Conc	Calculated Conc	Accuracy	Conc Mean	Mean Accuracy	Conc SD	Precision
	(µM)	(µM)	%	(µM)	%	(µM)	RSD %
5	0.15	0.1561	104.1				
5	0.15	0.1454	96.9	0.151	103.7	0.008	5.02
6	0.44	0.4672	106.2				
6	0.44	0.4771	108.4	0.472	99.0	0.007	1.48
7	1.33	1.4309	107.6				
7	1.33	1.3808	103.8	1.406	101.8	0.035	2.52
		**Mean Accuracy**	**104.5**				
		**±**	**4.2**				

**Table 3:** Plasma S1P Concentration Determination. Abbreviations: IS for internal standard; Conc for concentration; RT for retention time; SD for standard deviation; RSD for relative standard deviation; CV for coefficient of variance.

	Sample	RT (min)	Response (area)		Calculated			
			S1P	IS	Conc (μM)	Mean (μM)	SD	CV (%)
1	S1P Plasma 1-1.d	4.502	16,799	12,098	1.05			
2	S1P Plasma 1-2.d	4.499	17,279	11,983	1.09			
3	S1P Plasma 1-3.d	4.498	16,837	12,520	1.02	1.05	0.04	3.46
4	S1P Plasma 2-1.d	4.502	15,446	13,537	0.87			
5	S1P Plasma 2-2.d	4.498	15,889	14,551	0.83			
6	S1P Plasma 2-3.d	4.496	16,723	13,915	0.91	0.87	0.04	4.75
7	S1P Plasma 3-1.d	4.499	16,739	11,792	1.08			
8	S1P Plasma 3-2.d	4.501	17,601	12,009	1.11			
9	S1P Plasma 3-3.d	4.493	17,945	13,172	1.03	1.07	0.04	3.61
10	S1P Plasma 4-1.d	4.504	15,883	12,545	0.96			
11	S1P Plasma 4-2.d	4.503	16,393	12,439	1.00			
12	S1P Plasma 4-3.d	4.494	17,445	12,800	1.03	1.00	0.04	3.64
		**Mean (μM)**	16,748.25	12,780.08	1.00			
		**SD**	753.58	856.09	0.09			
		**RSD (%)**	4.50	6.70	8.93			
